# Supplementary material for: Can Socioeconomic, Health, and Safety Data Explain the Spread of COVID-19 Outbreak on Brazilian Federative Units?
Source: Int J Environ Res Public Health. 2020 Nov 30;17(23):8921. doi: 10.3390/ijerph17238921 (PMC7730726; doi:10.3390/ijerph17238921)
Supplement: Supplementary file 1 [file ijerph-17-08921-s001.pdf]

Can Socioeconomic, Health, and Safety Data Explain the Spread of COVID-19 Outbreak on Brazilian Federative Units?

Diego Galvan [1,2,3]*, Luciane Effting [4], Hágata Cremasco [4], Carlos Adam Conte-Junior [1,2,3]

[1] COVID-19 Research Group, Center for Food Analysis (NAL), Technological Development Support Laboratory (LADETEC), Cidade Universitária, Rio de Janeiro, RJ, 21941-598, Brazil; conte@iq.ufrj.br
[2] Laboratory of Advanced Analysis in Biochemistry and Molecular Biology (LAABBM), Department of Biochemistry, Federal University of Rio de Janeiro (UFRJ), Cidade Universitária, Rio de Janeiro, RJ, 21941-909, Brazil
[3] Nanotechnology Network, Carlos Chagas Filho Research Support Foundation of the State of Rio de Janeiro (FAPERJ), Rio de Janeiro, RJ, 20020-000, Brazil
[4] Chemistry Department, State University of Londrina (UEL), Londrina, PR, 86057-970, Brazil; luciane.effting@uel.br; hagata@uel.br
* Correspondence: diegogalvann@gmail.com; diegogalvann@iq.ufrj.br, orcid: https://orcid.org/0000-0001-8394-3431.

Supporting Information

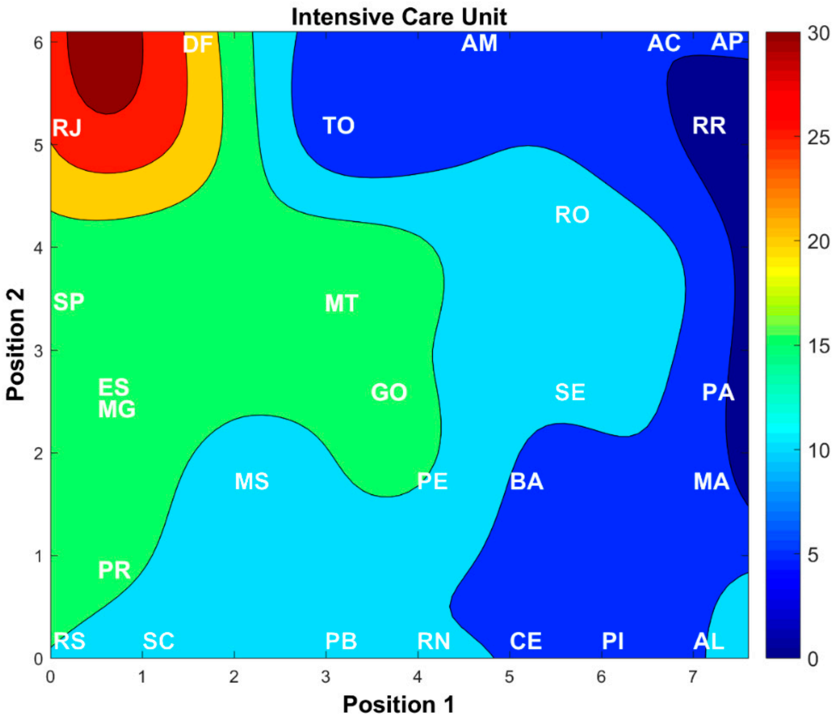

Figure S1 – Weight maps overlaid by topological maps for variable **Intensive Care Unit (ICU)** by Brazilian federative unit.

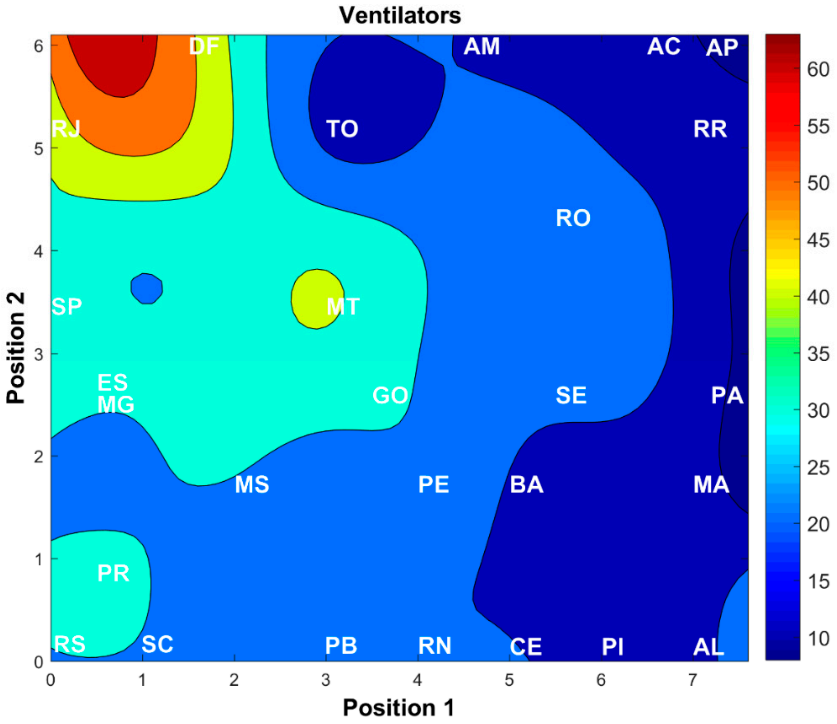

Figure S2 – Weight maps overlaid by topological maps for variable **Ventilators** by Brazilian federative unit.

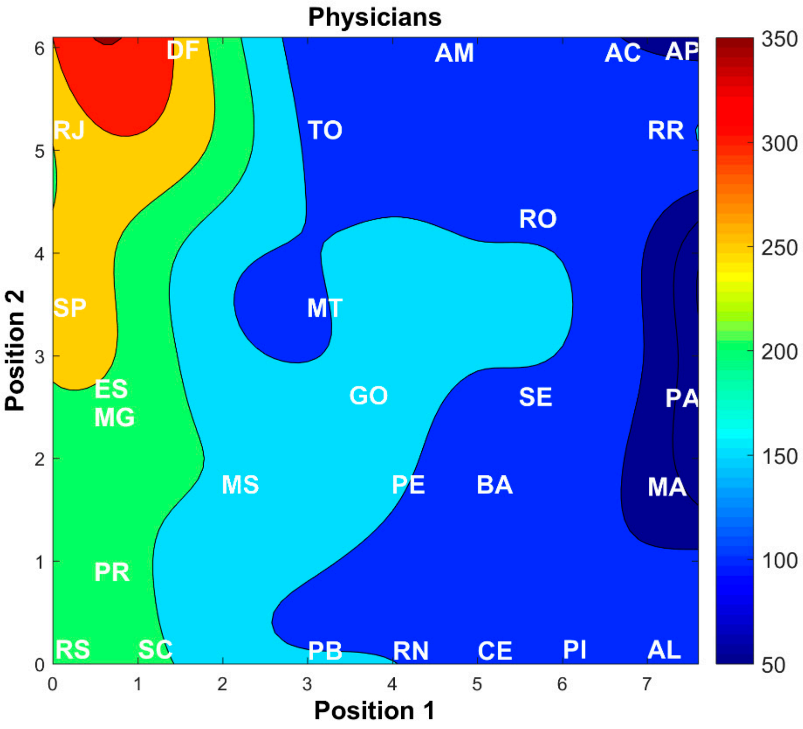

Figure S3 – Weight maps overlaid by topological maps for variable **Physicians** by Brazilian federative unit.

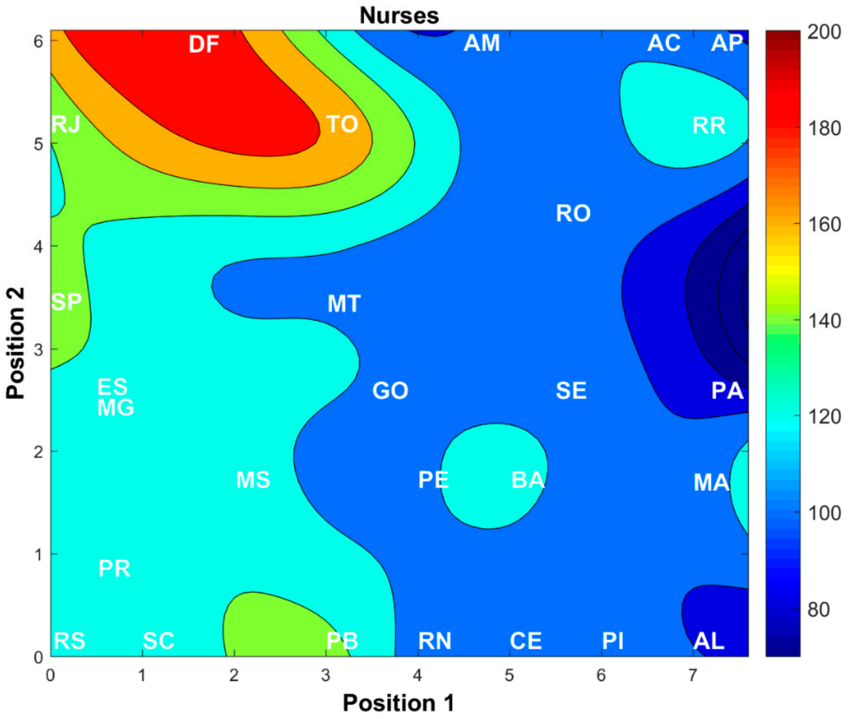

Figure S4 – Weight maps overlaid by topological maps for variable **Nurses** by Brazilian federative unit.

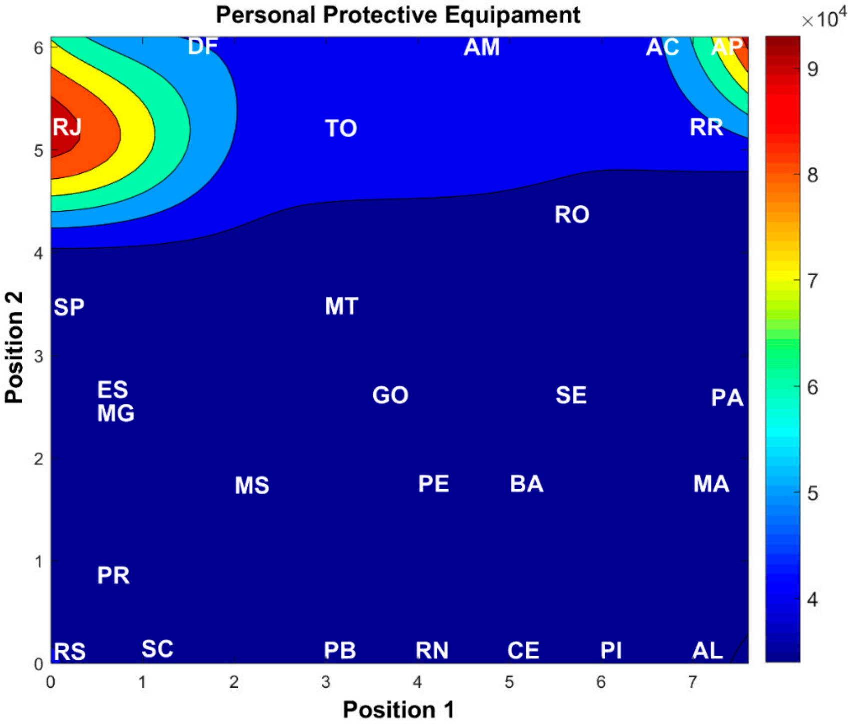

Figure S5 – Weight maps overlaid by topological maps for variable **Personal Protective Equipment** by Brazilian federative unit.

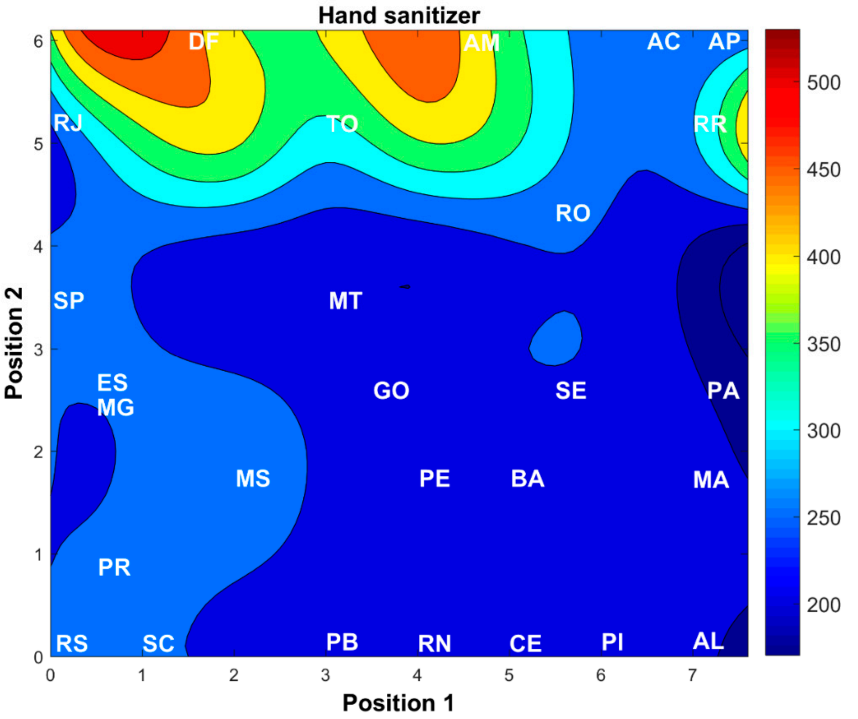

Figure S6 – Weight maps overlaid by topological maps for variable **Hand sanitizer** by Brazilian federative unit.

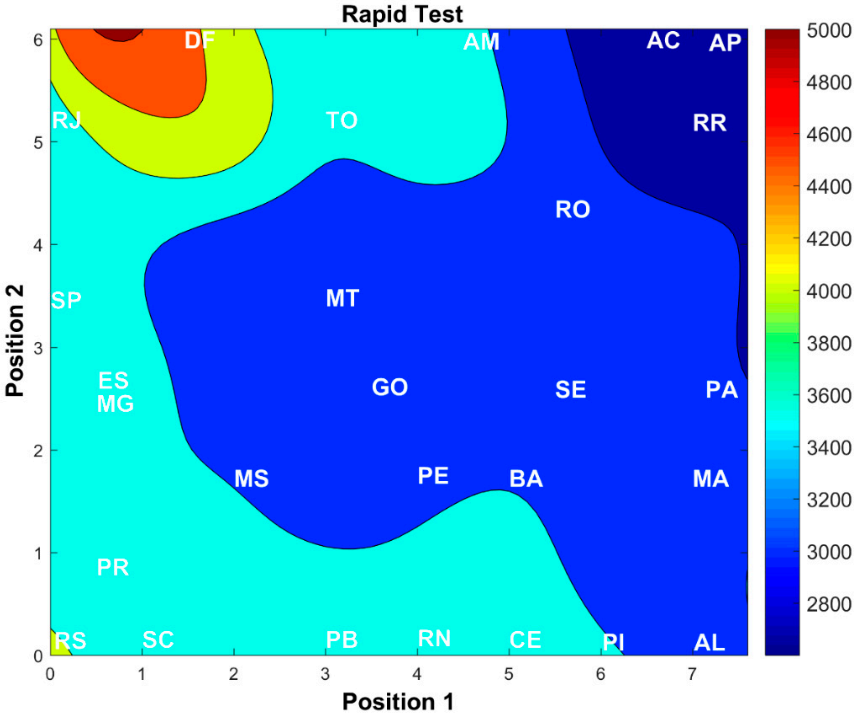

Figure S7 – Weight maps overlaid by topological maps for variable **Rapid test** by Brazilian federative unit.

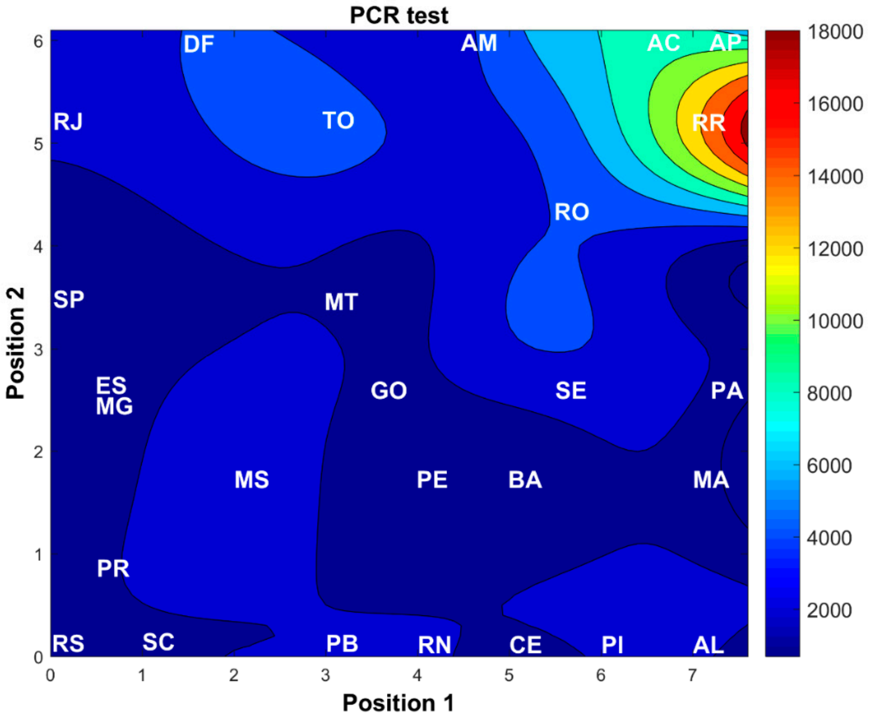

Figure S8 – Weight maps overlaid by topological maps for variable **PCR test** by Brazilian federative unit.

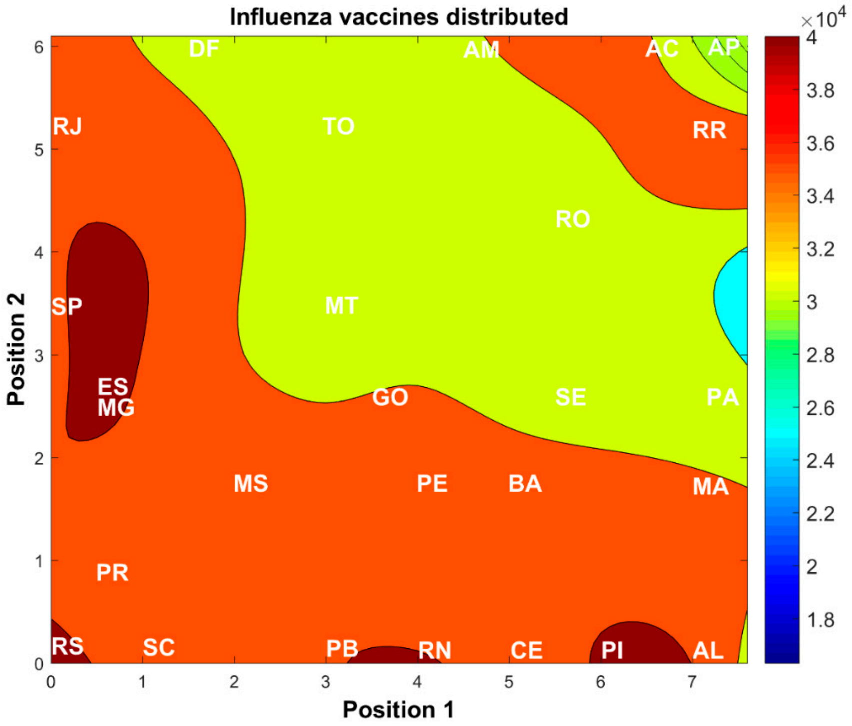

Figure S9 – Weight maps overlaid by topological maps for variable **Influenza vaccines distributed** by Brazilian federative unit.

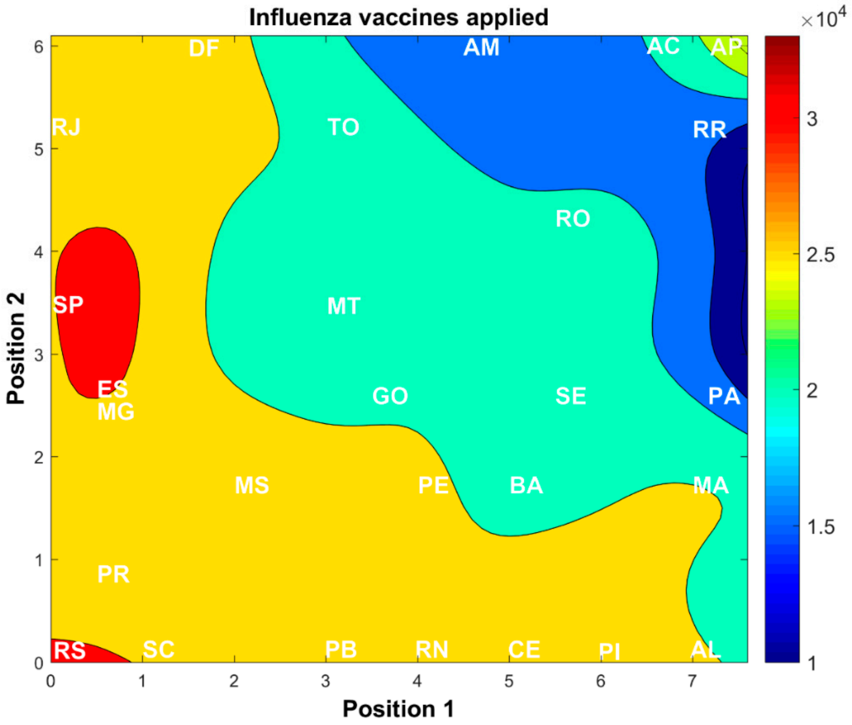

Figure S10 – Weight maps overlaid by topological maps for variable **Influenza vaccines applied** by Brazilian federative unit.

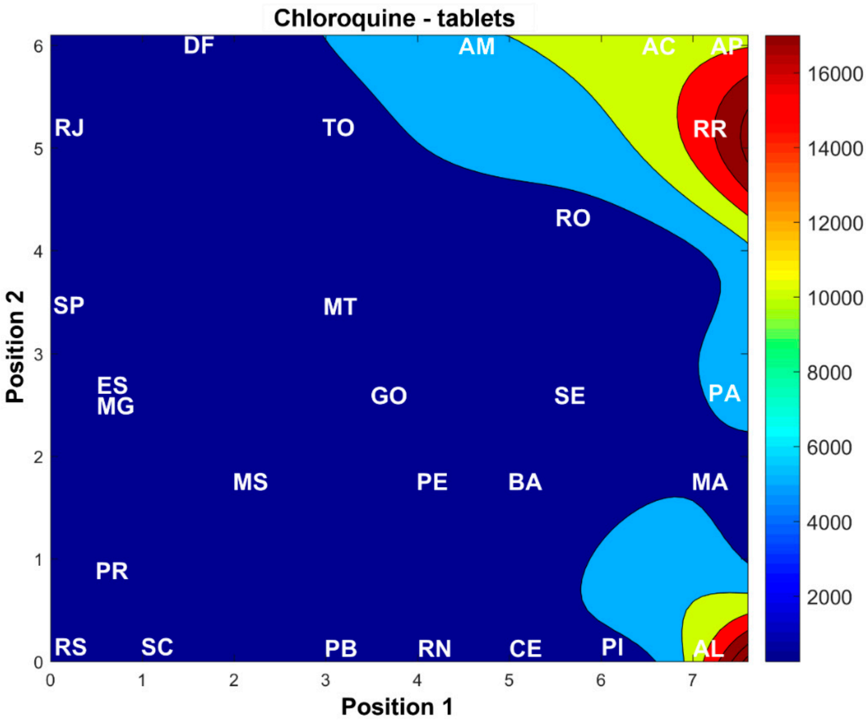

Figure S11 – Weight maps overlaid by topological maps for variable **Chloroquine tablets** by Brazilian federative unit.

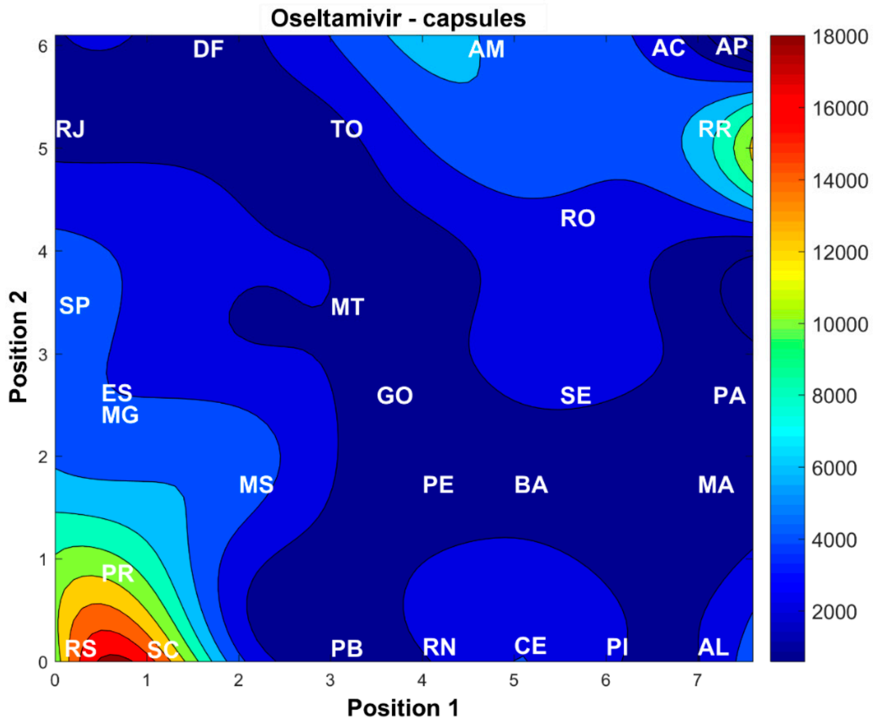

Figure S12 – Weight maps overlaid by topological maps for variable **Oseltamivir capsules** by Brazilian federative unit.

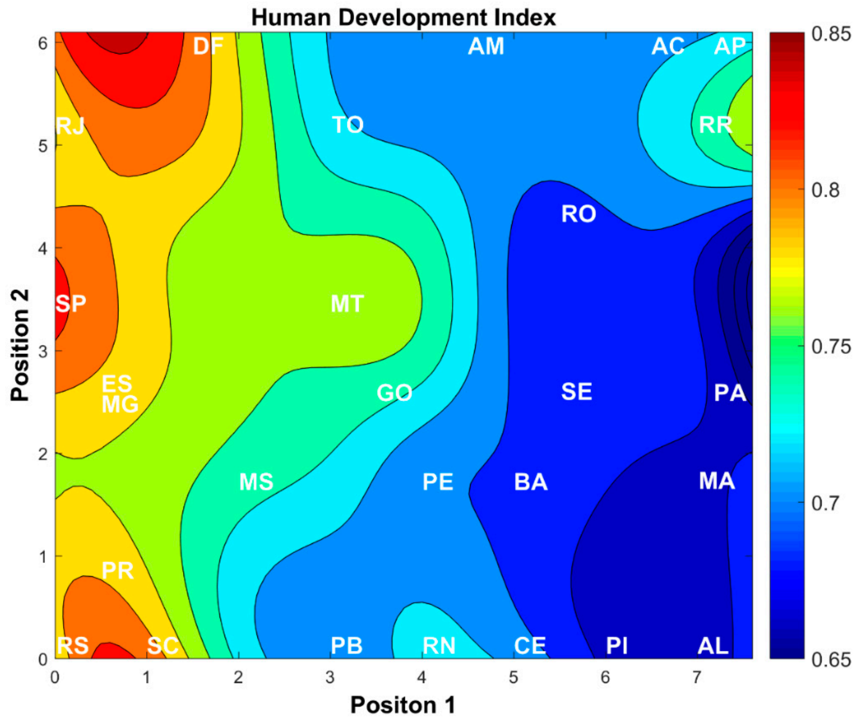

Figure S13 – Weight maps overlaid by topological maps for variable **Human Development Index (HDI)** by Brazilian federative unit.

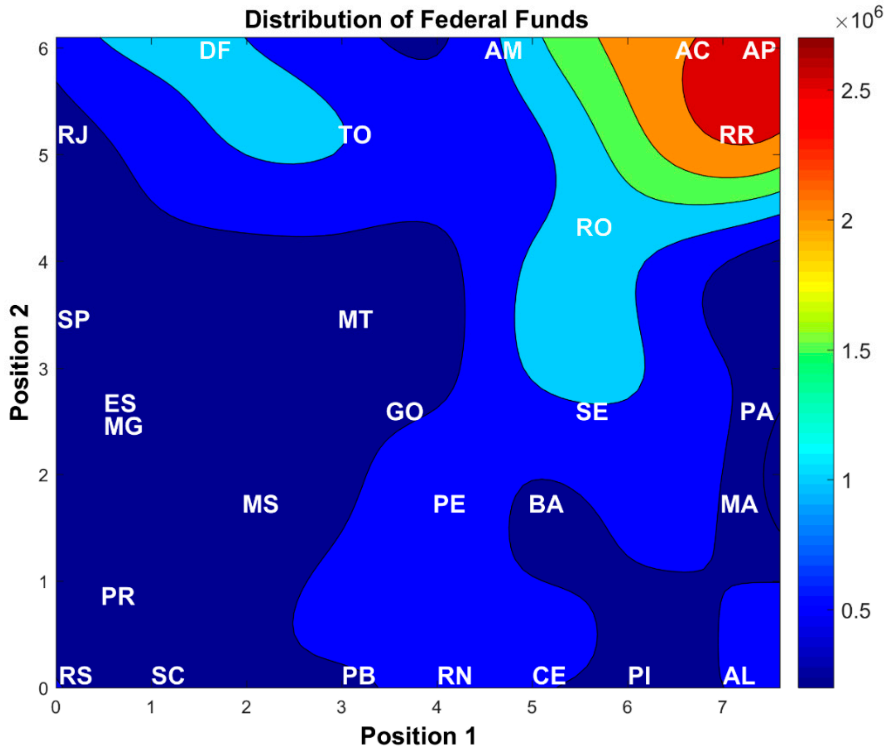

Figure S14 – Weight maps overlaid by topological maps for variable **Distribution of Federal funds** by Brazilian federative unit.

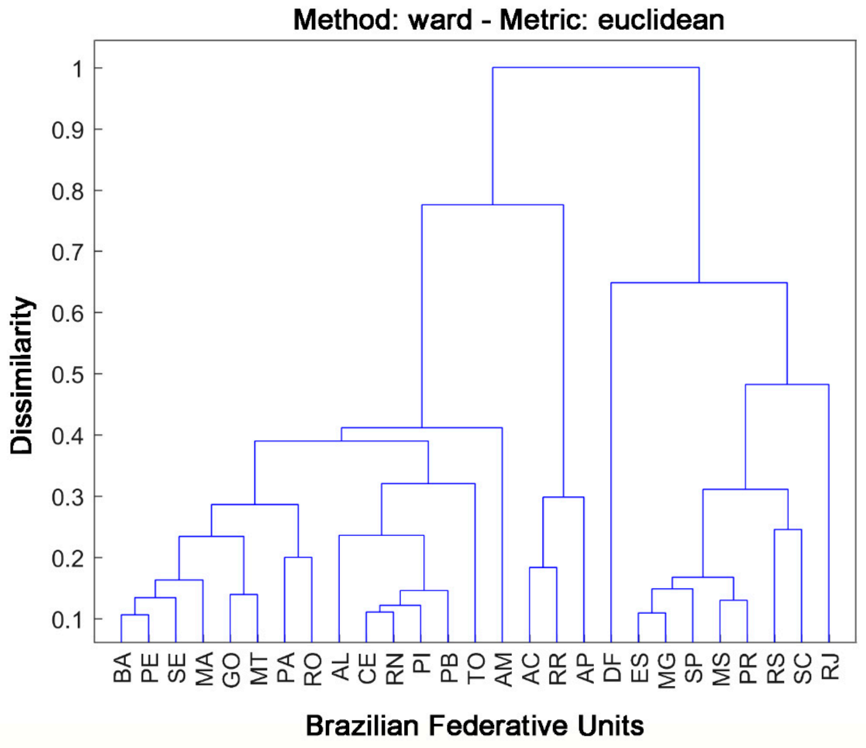

Figure S15 – Dendrogram of an HCA for Brazilian Federative Units.
